# Rapid diagnosis of *Talaromyces marneffei* infection by metagenomic next-generation sequencing technology in a Chinese cohort of inborn errors of immunity

**DOI:** 10.3389/fcimb.2022.987692

**Published:** 2022-09-08

**Authors:** Lipin Liu, Bijun Sun, Wenjing Ying, Danru Liu, Ying Wang, Jinqiao Sun, Wenjie Wang, Mi Yang, Xiaoying Hui, Qinhua Zhou, Jia Hou, Xiaochuan Wang

**Affiliations:** Department of Clinical Immunology, Children’s Hospital of Fudan University, National Children’s Medical Center, Shanghai, China

**Keywords:** *Talaromyces marneffei*, metagenomic next-generation sequencing, inborn errors of immunity, *IL12RB1* mutation, T-cell-mediated immunity, intrinsic and innate immunodeficiencies

## Abstract

*Talaromyces marneffei* (*T. marneffei*) is an opportunistic pathogen. Patients with inborn errors of immunity (IEI) have been increasingly diagnosed with *T. marneffei* in recent years. The disseminated infection of *T. marneffei* can be life-threatening without timely and effective antifungal therapy. Rapid and accurate pathogenic microbiological diagnosis is particularly critical for these patients. A total of 505 patients with IEI were admitted to our hospital between January 2019 and June 2022, among whom *T. marneffei* was detected in 6 patients by metagenomic next-generation sequencing (mNGS), and their clinical and immunological characteristics were summarized. We performed a systematic literature review on *T. marneffei* infections with published immunodeficiency-related gene mutations. All patients in our cohort were confirmed to have genetic mutations in *IL12RB1*, *IFNGR1*, *STAT1*, *STAT3*, and *CD40LG*. *T. marneffei* was detected in both the blood and lymph nodes of P1 with *IL12RB1* mutations, and the clinical manifestations were serious and included recurrent fever, weight loss, severe anemia, splenomegaly and lymphadenopathy, all requiring long-term antifungal therapy. These six patients received antifungal treatment, which relieved symptoms and improved imaging findings. Five patients survived, while one patient died of sepsis after hematopoietic stem cell transplantation. The application of mNGS methods for pathogen detection in IEI patients and comparison with traditional diagnosis methods were investigated. Traditional diagnostic methods and mNGS tests were performed simultaneously in 232 patients with IEI. Compared to the traditional methods, the sensitivity and specificity of mNGS in diagnosing *T. marneffei* infection were 100% and 98.7%, respectively. The reporting time for *T. marneffei* detection was approximately 26 hours by mNGS, 3-14 days by culture, and 6-11 days by histopathology. *T. marneffei* infection was first reported in IEI patients with *IL12RB1* gene mutation, which expanded the IEI lineage susceptible to *T. marneffei*. For IEI patients with *T. marneffei* infection, we highlight the application of mNGS in pathogenic detection. mNGS is recommended as a front-line diagnostic test for rapidly identifying pathogens in complex and severe infections.

## 1 Introduction

*Talaromyces marneffi* (*T. marneffei*), previously known as *Penicillium marneffei*, is an emerging pathogenic fungus causing fatal systemic mycosis in Southeast Asia, especially Thailand, northeastern India, Vietnam, southern China, Hong Kong and Taiwan (DiSalvo et al., 1973; Deng et al., 1988; Hu et al., 2013). The common manifestations of disseminated *T. marneffei* infection include recurrent fever, anemia, weight loss, skin lesions, hepatosplenomegaly, lymphadenopathy, and gastrointestinal and respiratory signs. When *T. marneffei* infection disseminates systemically, it can run a rapidly progressive course and be life-threatening without timely and effective antifungal therapy (You et al., 2021).

The vast majority of *T. marneffei* cases are diagnosed in HIV-positive patients (Supparatpinyo et al., 1994; Kawila et al., 2013), while cases have also been described in other immunocompromised individuals, such as patients with adult-onset acquired immunodeficiency due to autoantibodies against interferon-gamma (IFN-γ) (Guo et al., 2020), various inborn errors of immunity (IEI) (Tangye et al., 2020), hematological malignancies, diabetes mellitus and those taking corticosteroids or immunosuppressive agents (Qiu et al., 2015). In recent years, *T. marneffei* infection in children with IEI has been increasingly diagnosed. Genetic mutations involving *STAT1, STAT3, CD40LG, CARD9* and *IFNGR1* have been reported to be associated with *T. marneffei* infection (Ma et al., 2009; Lee et al., 2012; Du et al., 2019; Lee et al., 2019; Ba et al., 2021; You et al., 2021). However, as more cases are reported, other potential gene mutations may be discovered that predispose the host to *T. marneffei* infection.

Early identification and rapid diagnosis of *T. marneffei* infection are critical to a good prognosis. Traditional diagnostic methods of *T. marneffei* infection rely on culture or microscopy, which are time-consuming and have a lower positive rate. In recent years, the development of metagenomic next-generation sequencing (mNGS) has greatly improved the detection of pathogenic microorganisms (Chiu and Miller, 2019; Li et al., 2020; Qian et al., 2020; Chen et al., 2021; Su et al., 2022). mNGS can simultaneously accomplish the detection of bacteria, fungi, viruses, parasites and other pathogens with high efficiency and high sensitivity (Goldberg et al., 2015; Schlaberg et al., 2017). Currently, mNGS has matured into clinical applications in the diagnosis of complex and severe infections (Wang et al., 2020).

In this study, we reported the clinical and immunological characteristics of six patients with *T. marneffei* infection in a cohort of Chinese patients with IEI and emphasized the application value of mNGS in the diagnosis of *T. marneffei* infection. We aimed to improve the early recognition of *T. marneffei* infection in IEI patients. The immunodeficiency spectrum of *T. marneffei* infection was further broadened.

## 2 Methods

This study was approved by the Ethics Committee of the Children’s Hospital of Fudan University. Written informed consent was obtained from the parents of all patients.

### 2.1 Study design and patients

Patients with IEI admitted to the Children’s Hospital of Fudan University between January 2019 and June 2022 were enrolled consecutively, and patients with *T. marneffei* infection were retrospectively summarized.

Children with IEI were defined as patients previously assigned a diagnosis of IEI or patients admitted for abnormal clinical manifestations, including severe/unusual infections, and then diagnosed with IEI. The clinical manifestations, immune function, genetic testing, and pathogenic microorganisms were evaluated. Routine tests of peripheral blood, urine, stool and blood culture were performed in all patients. The cultures of sputum or bronchoscopic alveolar lavage fluid (BALF) were detected in patients with pulmonary infection. Further mNGS testing was performed in patients considered to have severe or unusual pathogen infection.

Definition of terms: The age at the last follow-up was defined as the age at July 2022 or the age at death. The diagnosis of bacterial infections was based on positive culture results. The (1–3) -β-d-glucan (G) tests were defined as positive when > 100 pg/ml (Lu et al., 2011; Li et al., 2015). The galactomannan (GM) index was defined as positive when > 0.5. The GM test is highly sensitive to Aspergillus infection (Guo et al., 2010). The diagnostic criteria for Bacillus Calmette Guerin (BCG) disease were described in a previous publication (Zhou et al., 2018). Epstein-Bar virus (EBV) and cytomegalovirus (CMV) infections were based on PCR detection of DNA. Disseminated *T. marneffei* infection was defined as infections involving more than two organs or systems.

### 2.2 Routine immune function evaluation

Routine blood counts and immunological function analyses were performed. We used nephelometry to detect immunoglobulins (Igs), including IgG, IgA, and IgM. Lymphocyte subsets were measured using flow cytometry (Becton Dickinson, Franklin Lakes, NJ, USA). The following validated antibodies were used for flow cytometry: anti-CD3 (UCHT1), anti-CD8 (RPAT8), anti-CD27 (M-T271), anti-CD45RA (HI100), anti-CD4 (RPA-T4), anti-TCRαβ (T10B9.1A-31), anti-TCRγδ (B1), anti-CD19 (HIB19), anti-CD24 (ML5), anti-CD38 (HIT2), and anti-IgD (IA6-2) (all from BD Biosciences) (Ding et al., 2018). Flow cytometry detection of CD119 and CD212 was performed.

### 2.3 Genetic analysis

Genomic DNA was extracted from the peripheral blood of the patients and their parents using the QIAamp DNA Blood Mini kit (Qiagen, Hilden, Germany). DNA quality was assessed using a NanoDrop ultraviolet spectrophotometer (Thermo Fisher Scientific, USA).

Next-generation sequencing was performed using a panel that included all previously reported immunodeficiency genes. Genomic DNA fragments of patients were ligated with adaptors so that two paired-end DNA libraries with insert sizes of 500 bp were formed for all samples. After enrichment, the DNA libraries were sequenced on the HiSeq 2000 platform in accordance with the manufacturer’s instructions (Illumina, San Diego, CA). The variants were annotated in ANNOVAR and VEP software and predicted with SIFT, PolyPhen-2 and MutationTaster.

### 2.4 Culture and identification of *T. marneffei*

*T. marneffei* was traditionally identified by culture or histopathology (Cao et al., 2019). In our study, *T. marneffei* was isolated from bacterial or fungal cultures of blood and BALF samples. This microorganism typically took approximately 3 to 14 days (Le et al., 2011; Cao et al., 2019). *T. marneffei* was identified by the following criteria (Segretain, 1959; Liyan et al., 2004; Cao et al., 2019) (1): Colonies of *T. marneffei* were verified by a MALDI-TOF mass spectrometer system (Bruker, Germany) (2). Positive fungal cultures were confirmed by Gram staining of a smear of the blood culture broth, followed by subculture onto Sabouraud dextrose agar (SDA) with incubation at 25 ˚C and 37 ˚C in room air. Yeast conversion was performed to confirm the identification of *T. marneffei*, and typical fungal colonies were observed (yeast phase at 37 ˚C with no red pigment production and mycelia at 25 ˚C with massive red pigment production) (3). Microscopically, the fungus had typical filamentous reproductive structures of the genus Penicillium, including the presence of conidiophore-bearing biverticillate penicilli, with each penicillus being composed of four to five metulae with smooth-walled conidia. *T. marneffei.* can be observed in histopathological sections, and methenamine silver or periodic acid-Schiff (PAS) staining was preferred. The presence of sausage-like cells, 2-3 μm in diameter, or elongated yeast-like organisms with clear central septum was the specific feature of *T. marneffei (* Liyan et al., 2004).

### 2.5 Metagenomic next-generation sequencing

#### 2.5.1 Nucleic acid extraction

Blood and lymph node biopsy tissue samples were collected according to standard procedures. Plasma was prepared from blood samples, and circulating cell-free DNA (cfDNA) was isolated from plasma with the QIAamp Circulating Nucleic Acid Kit (Qiagen) according to the manufacturer’s protocols. Lymph node biopsy tissue was extracted using the QIAamp DNeasy Blood & Tissue Kit (Qiagen) according to the manufacturer’s protocols. The quantity and quality of DNA were assessed using Qubit (Thermo Fisher Scientific) and NanoDrop (Thermo Fisher Scientific), respectively.

#### 2.5.2 Library preparation and sequencing

DNA libraries were prepared using the KAPA Hyper Prep kit (KAPA Biosystems) according to the manufacturer’s protocols. An Agilent 2100 was used for quality control, and DNA libraries were 75 bp single-end sequenced on an Illumina NextSeq 550Dx (Illumina).

#### 2.5.3 Bioinformatics analysis

Raw sequencing data were split by bcl2fastq2, and high-quality sequencing data were generated using Trimmomatic by removing low-quality reads, adapter contamination, duplicates and shot (length<36 bp) reads. Human host sequences were subtracted by mapping to the human reference genome (hs37d5) using Bowtie2. Reads that could not be mapped to the human genome were retained and aligned with the microorganism genome database for microbial identification by Kraken and for species abundance estimation by Bracken. The microorganism genome database contained genomes or scaffolds of bacteria, fungi, viruses and parasites (downloaded from GenBank release 238, ftp://ftp.ncbi.nlm.nih.gov/genomes/genbank/).

#### 2.5.4 Interpretation and reporting

We used the following criteria for positive mNGS results: For Mycobacterium, Nocardia and Legionella pneumophila, the result was considered positive if a species detected by mNGS had a species-specific read number ≥1. For bacteria (excluding Mycobacterium, Nocardia and Legionella pneumophila), fungi, viruses and parasites, the result was considered positive if a species detected by mNGS had at least 3 nonoverlapping reads. Pathogens detected in the negative ‘no-template’ control (NTC) were included only if the detected reads were ≥10-fold greater than those in the NTC.

### 2.6 Statistical analysis

Data were analyzed using SPSS 26.0 (IBM Corp., Armonk, NY, USA). Categorical variables were displayed as numbers and percentiles. The paired McNemar chi-square test was used to analyze the diagnostic efficiency of mNGS vs. conventional culture methods. Two-sided P values < 0.05 were considered statistically significant.

## 3 Results

From January 2019 to June 2022, a total of 505 cases of IEI were admitted to our hospital (**Table 1**). One hundred and three (20.4%) patients were diagnosed with IEI before admission, while 402 (79.6%) patients were definitively diagnosed with IEI after admission. Most of the patients (425/505) were admitted with various infectious manifestations. All patients underwent routine blood cultures. Seventy-three patients underwent histopathology. Nearly half of the patients (232/505) underwent further mNGS testing. Therefore, 232 patients (45.9%) underwent both culture and mNGS detection.

**Table 1:** Baseline of patients with IEI in our cohort.

		Number of cases
Total patients		505
novelty diagnosed IEI		402
admitted due to infections		425
Culture		505
	blood stream	505
	sputum	260
	BALF	142
	bone marrow	10
	hydrothorax	1
	ascites	0
	blood+BALF	142
	blood+sputum	260
Histopathology		73
	lymph node	39
	colon	46
	liver	11
	bronchial mucosa	4
	skin	12
	bone	4
mNGS		232
	blood	131
	sputum	32
	BALF	131
	bone marrow	1
	hydrothorax/ lung abscess	3
	ascites	0
	biopsy	36
	lymph node	17
	liver	10
	lung	3
	skin	4
	bone	2
	blood+BALF	53
	blood+sputum	21
Culture + mNGS simultaneously		232
	blood	131
	BALF	131
	sputum	32
Histopathology + mNGS simultaneously		33
	lymph node	17
	liver	9
	lung	3
	skin	2
	bone	2

**Figure 1:**
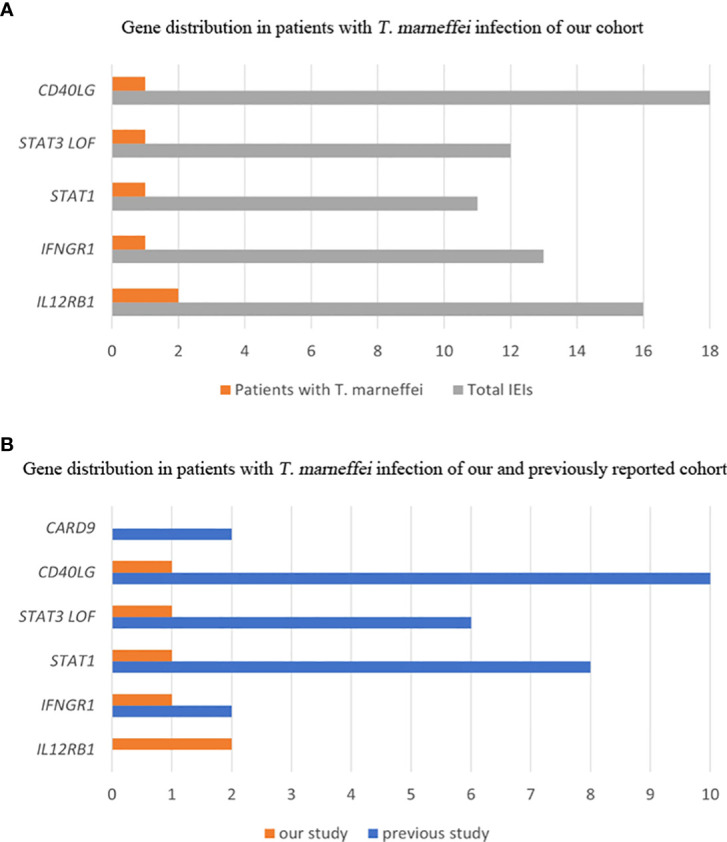
Gene distribution of patients with *T. marneffei* infection in IEIs. Gene distribution in our cohort **(A)**. Gene distribution in previously reported cases **(B)**.

*T. marneffei* infection was detected in six patients. The frequency of *T. marneffei* infection was 1.2% (6/505) in our cohort. The genetic mutations of *T. marneffei*-infected patients included *IL12RB1, IFNGR1, STAT3, STAT1* and *CD40LG* (**Figure 1A**). Detailed clinical (**Table 2**) and immunological (**Table 3**) information of each patient is described as follows.

**Table 2:** Clinical characteristics of T. marneffei infection in 6 patients with IEI.

	P1	P2	P3	P4	P5	P6
Gender	F	M	M	F	M	M
Province of residence	Hunan	Guangdong	Zhejiang	Jiangxi	Jiangxi	Guangdong
Age at diagnosis of IEI (m)	35m	5m	6m	12m	31m	7m
Age at T.marneffei infection (m)	36m	5m	29m	11m	29m	25m
Method of T. marneffei detection	mNGS of peripheral blood and lymph node biopsy tissue; blood culture; histopathology of lymph node	mNGS of peripheral blood	mNGS of BALF	mNGS and culture of BALF	mNGS of peripheral blood; histopathology of abdominal lymph nodes; blood culture	mNGS of peripheral blood and BALF
Infected site of T.marneffei	Blood, lymph node	Blood	Pulmonary	Pulmonary	Blood, lymph nodes, liver, spleen, pancreas	Blood, pulmonary
Clinical presentations						
Fever	+	-	-	+	+	+
Weight loss	+	-	-	-	NA	+
Anemia	+	-	+	+	+	+
Hepatomegaly	-	-	+	-	Multiple lesions of the liver	-
Splenomegaly	+	-	+	-	Multiple lesions of the spleen	Multiple lesions of the spleen
Lymphadenopathy	+	-	+	-	+	+
Bone destruction	-	-	+	-	-	-
Serous effusion	-	-	Pleural effusion	-	Pleural, peritoneal and pericardial effusion	-
Respiratory symptoms	Cough	-	-	Cough, shortness of breath, laryngeal stridor	Dysponea	_
Gastrointestinal symptoms	-	-	-	-	Recurrent nausea, diarrhea, vomiting, bloating	Diarrhea
Genetic tests	IL12RB1: c.875-885 deletion (paternal); exon 14-16 deletion (maternal)	IL12RB1: c.632G>C, p.R211P, hom	IFNGR1: c.655G>A, p.G219R, hom	STAT3: c.1394C>T, p.S465F, het	STAT1: c.821G>A, p.R274Q, het	CD40LG fragment deletion, hemi
Anti-antifungal Treatment	Itraconazole for 3 month, amphotericin B liposome for 2 weeks, then long-term itraconazole	Itraconazole for 6 months	Itraconazole for 9 months	Amphotericin B for 20 days, then long-term voriconazole	Intravenous voriconazole for 25 days, oral voriconazole for 7 months, then Itraconazole for 22 months	Itraconazole for 2 months
The last follow-up	Alive, 40m	Alive, 21m	Alive, 51m	Alive, 23m; HSCT at 18m	Alive, 68m	HSCT at 27m, died of sepsis at 29m

m, month; M, male; F, female; IEI, inborn error of immunity; BALF, bronchoalveolar lavage fluid; NA, Not Available; HSCT, hematopoietic stem cell transplantation.

**Table 3:** Hematological and immunological parameters at the time of T. marneffei infection in the six patients.

	P1	P2	P3	P4	P5	P6
WBC (´10^9^/L)	10.92	10.02	33↑	27↑	3.9↓	6.9
ANC (´10^9^/L)	7.73↑	1.74	24.72↑	13.15↑	1.94↓	4.38
ALC (´10^9^/L)	2.09↓	7.06	5.5	10.29↑	1.60↓	1.70↓
PLT (´10^9^/L)	188	402↑	680↑	662↑	40↓	353
Hb (g/L)	57↓	114	91↓	101↓	51↓	97↓
CRP (mg/L)	79↑	<8	79.8↑	8.7	20↑	46↑
ESR (mm/h)	125↑	2	120↑	NA	2	68↑
Ferritin (ng/mL)	256.6↑	60.5	51.3	NA	928.4↑	227↑
ALT (U/L)	6.22	17.1	16.2	21	26.1	45.9
AST (U/L)	18.5	42.5	28.6	38	136.9↑	63.1↑
G test (pg/mL)	271.89 ↑	<37.5	17.5	NA	1817.7 ↑	NA
GM test (blood/BALF)	6.682 ↑/NA	1.497 ↑/0.087	NA/0.120	NA/NA	6.192 ↑/NA	7.899↑/0.336
CD19 (cells/ul)	323.1 (18.60%)	1659.7 (27.54%)↑	439.7 (10.35%)↓	1734.9 (20.03%)	1304.6 (52.61%)↑	294.20 (15.95%)
Naive B (%)	84.02	NA	NA	89.62↑	96.30 ↑	97.7 ↑
Memory B (%)	7.89	NA	NA	2.76↓	0.10 ↓	0.2 ↓
Transitional B (%)	0.44↓	NA	NA	2.73↓	10.20	2.5↓
Plasmablasts (%)	0.41↓	NA	NA	0.29↓	0.20 ↓	0.80
CD3 (cells/ul)	1251.8 (72.05%)	4037.6 (66.99%)	3180.0 (74.88%)↑	5693.56 (68.86%)	945.7 (38.13%)↓	1452.3 (78.73%)
CD4 (cells/ul)	935.0 (53.82%)↑	2758.5 (45.77%)↑	2489.6 (58.62%)↑	3919.0 (45.25%)↑	628.4 (25.34%)↓	1034.72 (56.09%)
CD4 Naive (%)	58.87	NA	NA	74.20	51.80	85.30
CD4 CM (%)	34.85	NA	NA	23.40	45.60	14.30
CD4 EM (%)	6.21 ↑	NA	NA	2.25	2.60	0.4↓
CD4 TEMRA (%)	0.07	NA	NA	0.15	0.00	0.00
CD8 (cells/ul)	245.7 (14.14%) ↓	1177.6 (19.54%)↓	618.6 (14.57%)↓	1634.0 (18.87%)	247.92 (10%)↑	280.49 (15.21%)
CD8 Naive (%)	81.64	NA	NA	55.08	50.70	89.70
CD8 CM (%)	16.18	NA	NA	15.29	22.10	9.80
CD8 EM (%)	1.77	NA	NA	3.37	19.90 ↑	0.20
CD8 TEMRA (%)	0.42 ↓	NA	NA	26.26↑	7.20	0.30
DNT (%)	7.61	NA	NA	3.42	6.60	4.70
γδ T (%)	5.87	NA	NA	2.49↓	4.10 ↓	3.8↓
CD16CD56 (cells/ul)	118.7 (6.83%)	242.3 (4.02%)	533.4 (12.56%)	877.41 (10.13%)	199.4 (8.04%)↓	69.44 (3.76%)
IgG (g/L)	53.00↑	9.40↑	21.80↑	13.56↑	19.40 ↑	1.00 ↓
IgM (g/L)	1.89	0.32↓	2.35↑	1.06	0.85	0.58 ↓
IgA (g/L)	0.60	0.09↓	1.21↑	0.70	0.84↑	0.02 ↓
IgE (KU/L)	49.46	16.12	142.11↑	548.00↑	18.14	9.81
The reference of immunoglobulin:						
	1-3m	4-6m	12-36m			
IgG (g/L)	2.75-7.50	3.7-8.3	5.52-11.46			
IgM (g/L)	0.05-0.60	0.14-0.5	0.06-0.74			
IgA (g/L)	0.10-0.70	0.33-1.25	0.6-2.12			
IgE (KU/L)	<100	<100	<100			

WBC, white blood count; ALC, absolute lymphocyte count; ANC, absolute neutrophil count; HB, haemoglobin; CM, central memory; EM, effector memory; TEMRA, terminal effector memory cytotoxic T cells; DNT, TCR αβ+ CD4 and CD8 double-negative T cell; NA, not available.

The percentage and numbers of lymphocyte subsets in the peripheral blood reference to the literature (Ding et al., 2018). ↑: higher than the normal value; ↓: lower than the normal value.

### 3.1 Case description

#### 3.1.1 Patient 1

**Figure 2:**
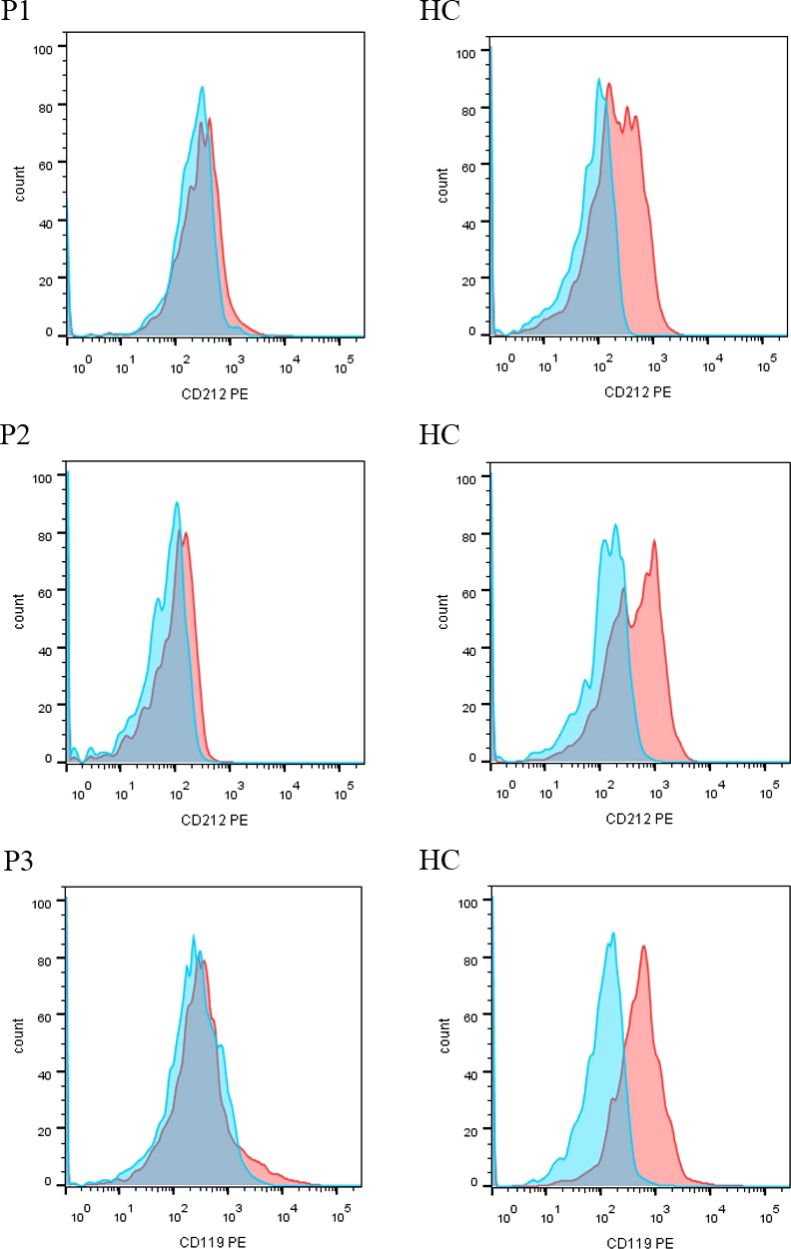
The expression of IL12RB1 (CD212) and IFNGR1 (CD119) protein in P1-P3. IL12RB1 protein was not expressed in P1 and P2. IFNGR1 protein was not expressed in P3.

**Figure 3:**
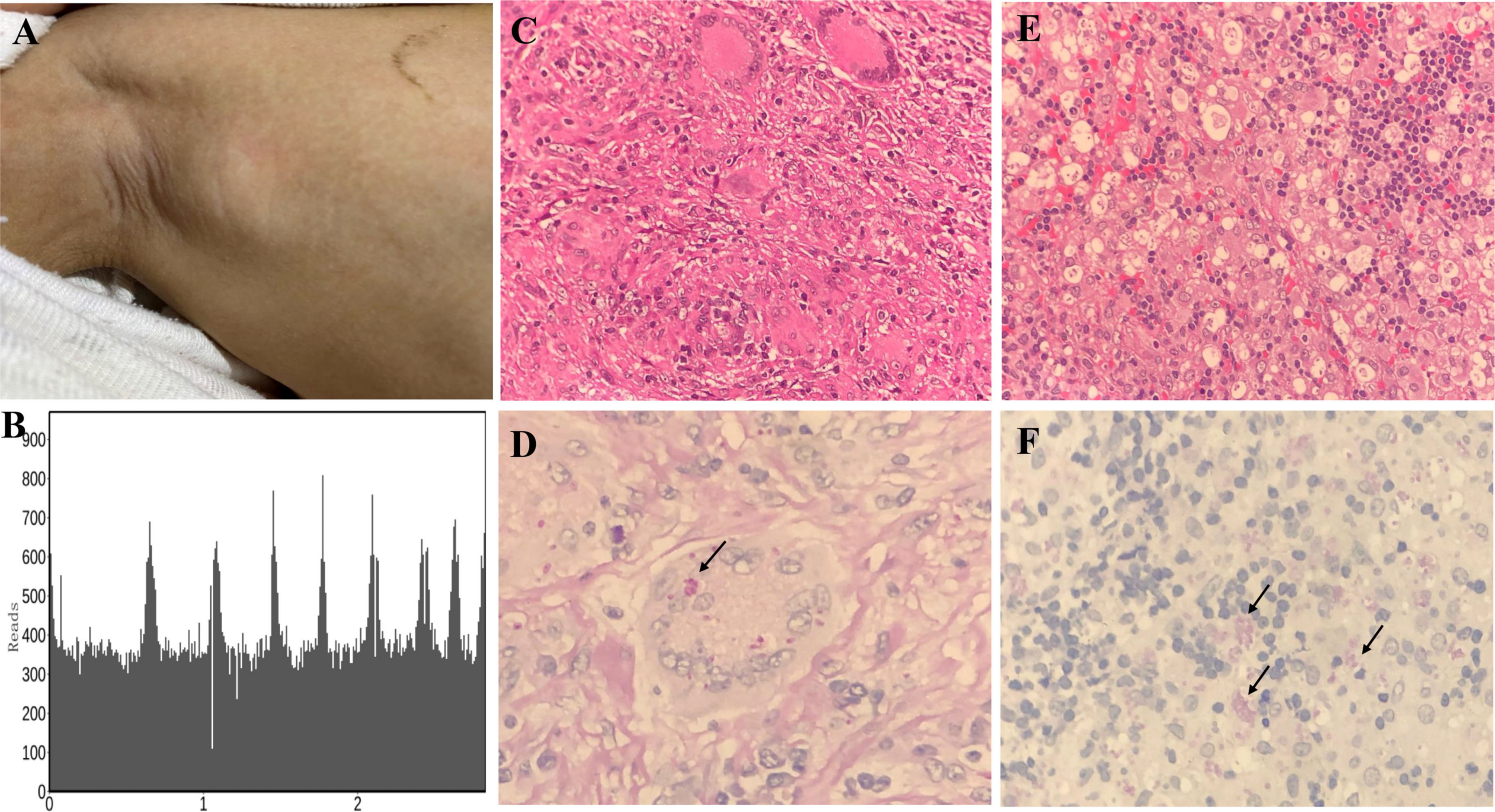
Histopathological staining and mNGS results in P1 and P5. Lymph node enlargement of the right axilla was seen in P1 **(A)**. Confirmation of T. marneffei-specific amplification from lymph node tissue by mNGS showed 108,380 unique sequence reads of T. marneffei, accounting for 18.93% of the genome coverage **(B)**. Granulomatous inflammation observed during histopathological examination of the cervical lymph node **(C)**. PAS staining of the cervical lymph node revealed fungal spores (arrows) (magnification × 400) **(D)**. A large number of neutrophil infiltrates were observed in the histopathological examination of abdominal lymph nodes of P5, and a patchy distribution of tissue cells was observed. Fungal spore-like substances were scattered or clustered in some tissue cells (magnification × 400) **(E)**. PAS staining of the abdominal lymph node tissues revealed fungal spores (arrows) (magnification × 400) **(F)**.

**Figure 4:**
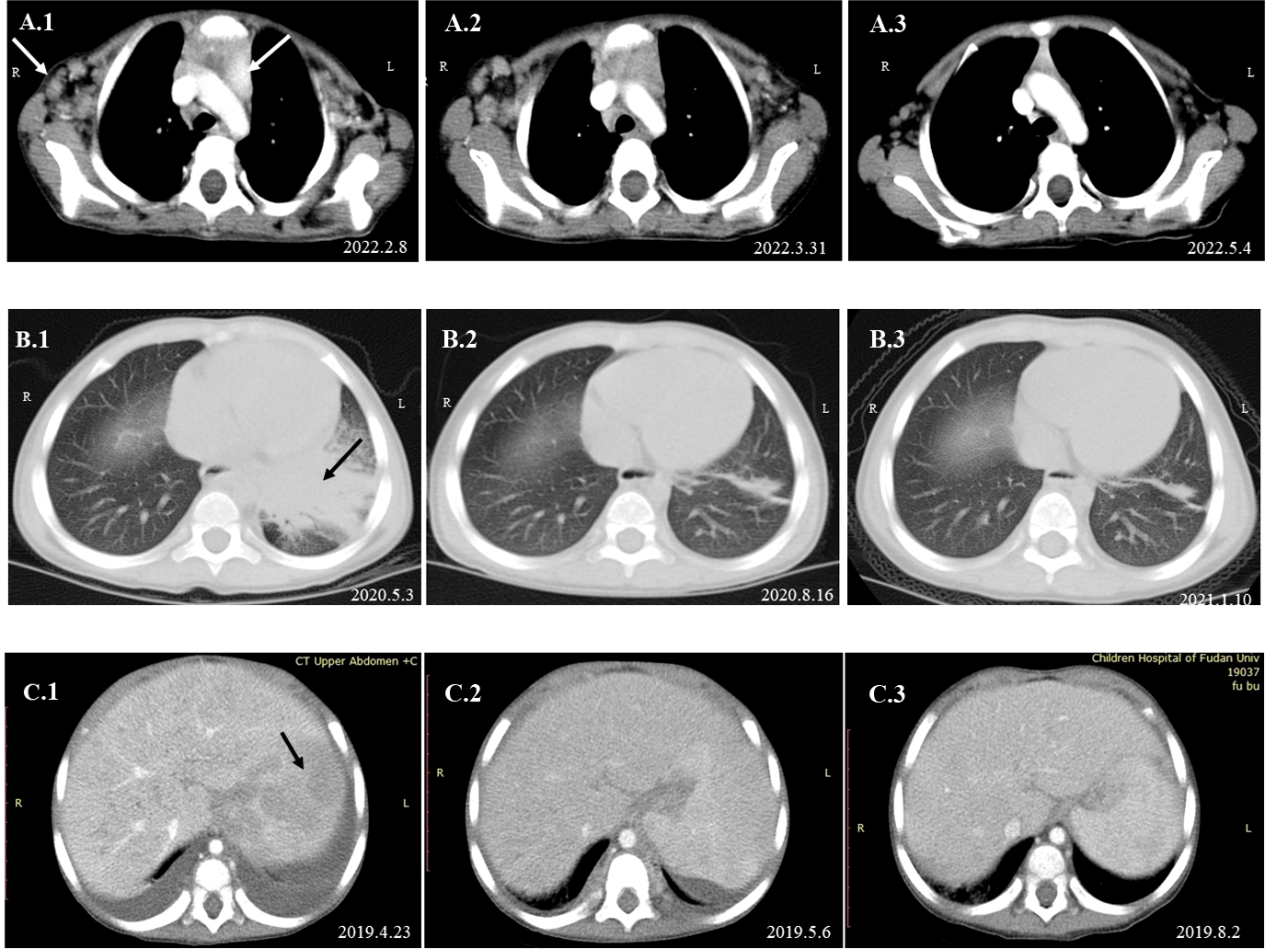
Dynamic changes in imaging examinations of patients during follow-up. Imaging examination of P1 revealed that the axillary and mediastinal lymph nodes were enlarged **(A.1)**. After oral itraconazole and anti-tuberculous therapy for nearly 2 months, chest CT re-examination showed that the axillary and mediastinal lymph nodes were still enlarged **(A.2)**. A lymph node biopsy was performed on April 19, 2022, and mNGS indicated high reads of T. marneffei. Antifungal therapy was adjusted to amphotericin B for 2 weeks, followed by oral itraconazole. One month later, the imaging examination suggested that the lymph nodes were smaller than before **(A.3)**. Chest CT of P3 suggested pneumonia and partial consolidation of the left lung with slight pleural effusion in the acute phase **(B.1)**, and the patient received oral itraconazole antifungal combined with anti-tuberculosis therapy. Chest CT re-examination revealed significant improvement in the lungs after 3 months **(B.2)** and 8 months **(B.3)**. Abdominal CT of P5 indicated hepatosplenomegaly and multiple abnormal lesions at the beginning **(C.1)**. He received intravenous voriconazole treatment for 25 days and then oral voriconazole. The multiple abnormal lesions improved after treatment for 2 weeks **(C.2)** and 3.5 months **(C.3)**.

A 3-year-old girl was admitted to our hospital with the chief complaint of lymphadenopathy and intermittent fever. The girl was vaccinated with BCG after birth, and she developed left subaxillary and cervical lymph node enlargement at the age of 3 months. The local hospital performed lymph node resection and administered anti-tuberculosis treatment for 6 months. Nine months after drug withdrawal, the cervical lymph node enlargement recurred, and anti-tuberculosis therapy was administered again. *IL12RB1* complex heterozygous mutations were detected: exon 14-16 deletion (maternal) and c.875-885 deletion (paternal). The expression of IL12RB1 protein was impaired (**Figure 2**). At the age of 3, the child developed recurrent fever, severe anemia, hepatosplenomegaly, and lymphadenopathy (**Figure 3A**), and the blood culture suggested *T. marneffei*. The serum G test (271.89 pg/ml) and GM test (6.682) were significantly increased. *T. marneffei* was detected in the blood (reads, 14) and lymph nodes (reads, 108380) by mNGS (**Figure 3B**). Therefore, isoniazid, rifampicin and ethambutol were used for antituberculosis, itraconazole for antifungal, and IFN-γ for immune regulation. The erythrocyte sedimentation rate (ESR) (120 mm/H) and G test (112.6 pg/ml) remained high after 2 months of anti-infective therapy, despite negative pathogens in blood by mNGS. Imaging examination revealed that the lymph nodes were still enlarged (**Figure 4A2**), and the liver and spleen lesions were smaller than before. After nearly 3 months of treatment with itraconazole, the biopsy of cervical lymph nodes suggested granulomatous inflammation (**Figure 3C**). PAS staining of the cervical lymph node tissues revealed fungal spores (**Figure 3D**), and *T. marneffei* was still identified by mNGS (reads, 15545702). Antifungal therapy was adjusted to amphotericin B liposomes intravenously for 2 weeks, followed by oral itraconazole. The follow-up CT scans revealed that the lymph nodes were obviously shrunken (**Figure 4A3**). ESR and G tests were reduced to normal, and then she was discharged.

#### 3.1.2 Patient 2

A 5-month-old boy (P2) was referred for enlarged axillary lymph nodes and ulceration of the BCG vaccination site. From 3 months of age, the child presented with left axillary lymph node enlargement and suppuration at the BCG vaccination site. Lymph node dissection and abscess removal were performed at 4 months of age. Acid fast bacilli were seen on pus smear, and Xpert examination of focal tissue suggested *Mycobacterium tuberculosis*. *Mycobacterium tuberculosis* (reads, 124) and *Escherichia coli* (reads, 952090) were detected in stool by mNGS. The child began to receive anti-tuberculosis treatment with isoniazid, rifampicin and ethambutol. At the age of 5 months, his lung CT indicated pulmonary cavities, and *Mycobacterium tuberculosis* was detected in BALF by mNGS. Levofloxacin was further added. In addition, the two blood GM test results were 0.589 and 1.497, respectively. Two mNGS tests of blood were performed 9 days apart, both of which were positive for *T. marneffei* (reads 29 and 6), and itraconazole was used for antifungal treatment. Whole-exome sequencing revealed a homozygous mutation of the *IL12RB1* gene, c.632G>C, p. R211P, derived from his parents. IL12RB1 protein was not expressed (**Figure 2**). He received oral itraconazole for 6 months with a good response and has continued anti-tuberculosis treatment to date.

#### 3.1.3 Patient 3

This male child was hospitalized for pneumonia during the neonatal period and was admitted with sepsis and liver dysfunction at the age of 2 months. At 3 months of age, he developed enlarged left axillary lymph nodes, redness, swelling and ulceration at the BCG vaccination site. The PPD test was strongly positive, and lymph node biopsy suggested granulomatous lesions. Isoniazid, rifampicin and ethambutol were used as anti-tuberculosis therapy, and IFN-γ was given as immunotherapy. Further genetic analysis indicated a homozygous mutation in the *IFNGR1* gene: c. 655G>A, p.G219R. IFNGR1 protein was confirmed not expressed (**Figure 2**). His parents discontinued medication on their own. The child developed leg pain at 24 months old and mandibular swelling 2 months later. Imaging examination suggested multiple bone destruction. The anti-tuberculosis treatment was performed again. He had recurrent fever, and lung CT suggested pneumonia and partial consolidation of the left lung (**Figure 4B1**). *T. marneffei* (reads, 15) was detected in BALF by mNGS, and then itraconazole was added for antifungal treatment for 9 months. After 3 and 8 months of antifungal treatment, chest CT revealed significant improvement (**Figures 4B2, 4B3**).

#### 3.1.4 Patient 4

The girl presented with recurrent skin eczema with pruritus from 1 month after birth. At the age of 9 months, the child developed low fever with a peak temperature of 38°C, accompanied by cough and expectoration, and pulmonary CT suggested bronchopneumonia. Routine blood examination revealed eosinophils of 1.58 × 10^9^/L and IgE raised to 548 IU/ml. The BALF was positive for *T. marneffei* by culture and mNGS (reads, 43) and CMV DNA. The GM test of the BALF was not tested at that time. She was treated with amphotericin B for 20 days, followed by voriconazole. Fever and cough recurred, and fibrobronchoscopy was performed again at 14 months. BALF indicated an elevated result of the GM test (1.335), and *Staphylococcus aureus* (reads, 42) and *Streptococcus pneumoniae* (reads, 14) were detected by mNGS. Voriconazole and linezolid were given for anti-infective therapy, and regular intravenous immunoglobulin (IVIG) was recommended. Gene testing suggested a *STAT3* gene heterozygous mutation: c.1394 C>T, p. S465F. With an NIH score of 39 (Grimbacher et al., 1999; Grimbacher et al., 1999), she was diagnosed with hyper IgE syndrome. Hematopoietic stem cell transplantation was performed at 18 months of age. She was in good condition during the six-month follow-up after transplantation.

#### 3.1.5 Patient 5

The patient developed recurrent nausea at 27 months of age without an obvious cause. Two months later, the symptoms worsened, with lethargy and fever. The G test was elevated at 1817.7 pg/ml, and abdominal CT indicated hepatosplenomegaly and multiple abnormal lesions (**Figure 4C1**). Routine blood tests suggested anemia and thrombocytopenia, while elevated levels of amylase and lipase suggested pancreatitis. Multiple lymph nodes were enlarged throughout the body, and histopathological examination of the abdominal lymph node biopsy presented a large number of neutrophil infiltrates and fungal spores in the tissue cells (**Figures 3E, F**). *T. marneffei* was further detected in blood by culture and mNGS (reads, 1452), and voriconazole was used for antifungal treatment. In addition, recurrent thrush began at the age of 1 year old, and genetic testing was performed, which revealed one *de novo* heterozygous mutation in the *STAT1* gene: c.821G>A, p.R274Q. After voriconazole treatment for 3 months, *T. marneffei* was negative in blood by mNGS. The multiple abnormal lesions in the liver and spleen improved (**Figure 4C3**). Then, he took itraconazole as a preventive antifungal treatment. Now the child has stopped antifungal drugs and is generally being well.

#### 3.1.6 Patient 6

The male patient presented with left axillary lymph node enlargement at 2 months of age, followed by supraclavicular lymph node enlargement. The 4-month-old child developed poor appetite, and *Pneumocystis jiroveci* was further detected in sputum and blood. The G test results significantly increased to 767-2242 pg/ml. Isoniazid and rifampicin were given for anti-tuberculosis treatment, sulfamethoxazole (SMZ) for *Pneumocystis jiroveci*, linezolid for bacteria, and itraconazole for anti-fungal treatment. According to his clinical presentations, he was considered to have an IEI, and his genetic test result was hemizygous *CD40LG* deletion. The child developed recurrent diarrhea and fever at the age of 2 after his parents stopped the medications on their own. Lung CT suggested exudation in both lungs. Abdominal MRI suggested abnormal signals in the spleen and both kidneys, multiple small lymph nodes in the abdominal cavity and ascites. *T. marneffei* (reads, 31) was detected in blood, and *T. marneffei* (reads, 58) and *Pneumocystis jiroveci* (reads, 4) were detected in BALF. Gastroenteroscopy showed ulceration of rectal, colonic, and small intestinal ulcers. Cefoperazone sulbactam, metronidazole, itraconazole, and SMZ were used as anti-infection treatments, and mesalazine was used as an inhibitor of intestinal inflammation. He died of sepsis after hematopoietic stem cell transplantation.

### 3.2 Diagnostic methods and sample types

A statistical study was performed in 232 patients who underwent both traditional standard and mNGS tests simultaneously (**Table 4**). We defined the patient as a positive case if *T. marneffei* was detected, regardless of specimen type. Traditional gold standard tests were culture and histopathology. Both mNGS and traditional methods were positive in 3 patients and negative in 226 patients. *T. marneffei* was detected only by mNGS in 3 patients but was negative by traditional methods. Next, we evaluated the diagnostic accuracy of mNGS in detecting *T. marneffei* infection. The sensitivity, specificity, positive likelihood ratio, and negative likelihood ratio of mNGS in our cohort were 100%, 98.7%, 76.9, and 0, respectively. McNemar chi-square analyses showed that there was no significant difference between the two methods (p=0.25).

**Table 4:** Diagnostic accuracy analysis of mNGS compared to traditional diagnostic methods.

	Tranditonal diagnostic methods +	Tranditonal diagnostic methods -	Total
mNGS +	3	3	6
mNGS -	0	226	226
Total	3	229	232

Sensitivity = 100%, Specificity = 98.7%, LR+ = 76.9, LR - = 0. LR, likelihood ratio.

**Figure 5:**
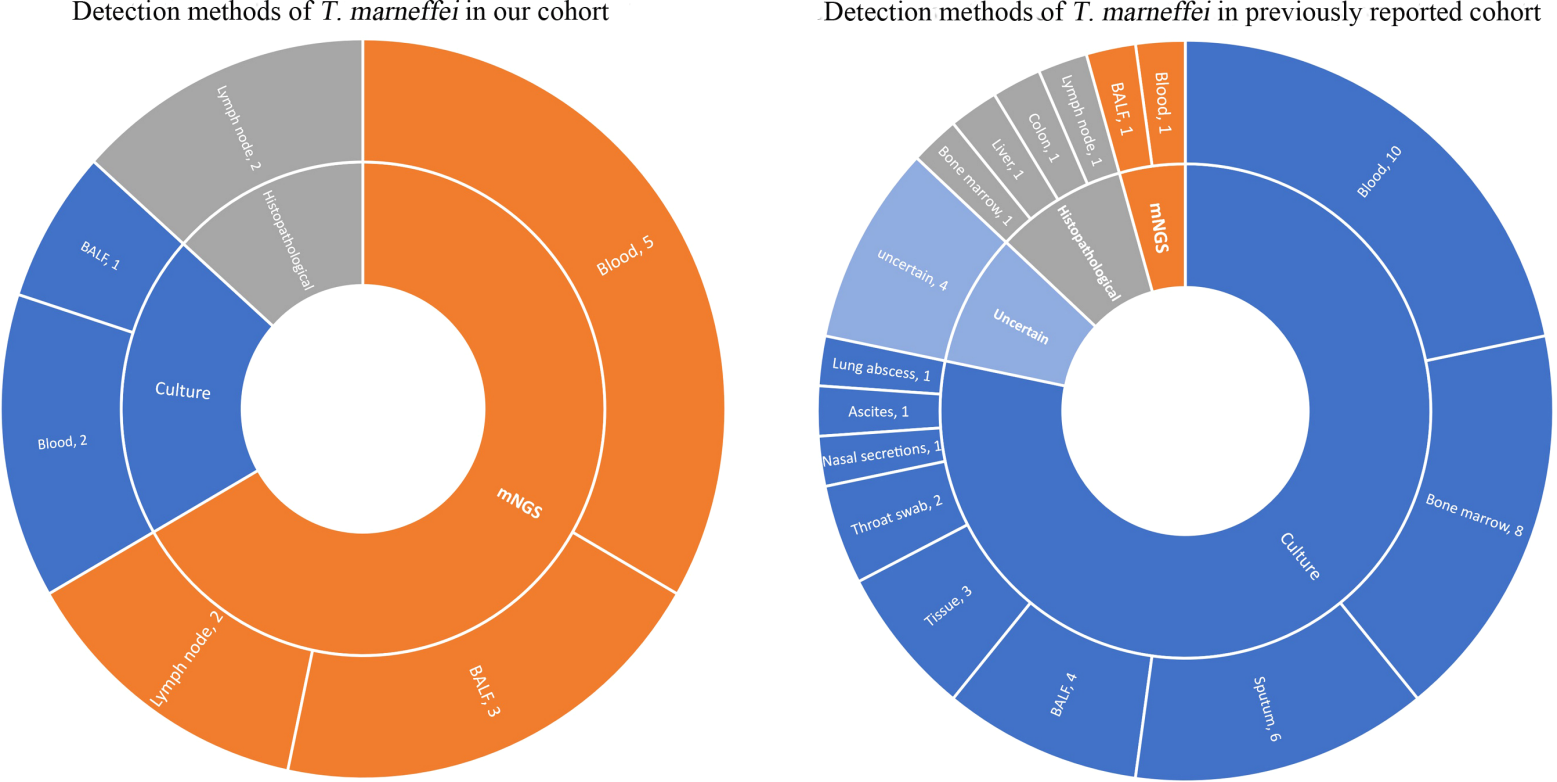
Distribution of sample types with T. marneffei positivity. In our cohort, T. marneffei was detected in 15 samples using different methods, including mNGS (n=10), culture (n=3), and histopathological staining (n=2). In the previous cohort, T. marneffei was mainly detected by cultures (n=36) and histopathological staining (n=4), and only two patients were detected by mNGS (n=2).

The sample detection methods of these 6 patients with *T. marneffei* infection were analyzed in detail. *T. marneffei* was identified in a total of 15 samples from six patients (**Figure 5**). In our cohort, *T. marneffei* was most commonly detected by mNGS, covering 5 blood samples, 3 BALF samples, and 2 lymph node samples. Only two blood samples and one BALF sample were identified by culture. The histopathological examination of the lymph nodes in two samples indicated the presence of *T. marneffei* infection. Culture and mNGS were performed on 12 samples from these six patients simultaneously. *T. marneffei* was identified in 2 samples by the culture method (16.7%, 2/12) and 8 samples by the mNGS method (66.7%, 8/12). One biopsy sample of a cervical lymph node was tested by histopathological staining and mNGS simultaneously. *T. marneffei* was identified with mNGS (reads, 15545702), and the histopathological PAS staining was also positive.

The time required for diagnosis using different methods for all six patients was further analyzed. The median reporting time for mNGS was 26 hours, with a range of 21.5-30 hours. The cultural identification periods of *T.marneffei* in the three positive samples were 3, 10 and 14 days. Two positive lymph node pathologic results were reported within 6 and 11 days. Therefore, the time consumption of mNGS was much less than that of culture or histopathology.

### 3.3 Literature review

A systematic literature review was performed in PubMed on human *T. marneffei* infections published between 1950 and 2022 using the key words ‘‘*Talaromyces marneffei*’’ or ‘‘*Penicillium marneffei*’’ or ‘‘*Penicilliosis*’’ or ‘‘*Talaromycosis*’’. Articles reporting *T. marneffei*-infected patients with immune-related gene mutations were included. HIV-positive cases were excluded. As a result, a total of 28 cases with confirmed IEI from seventeen articles were analyzed. The characteristics of the patients are summarized in **Table 5** (Yuen et al., 1986; Kamchaisatian et al., 2006; Ma et al., 2009; Sripa et al., 2010; Lee et al., 2012; Lee et al., 2014; Fan et al., 2018; Li et al., 2018; Du et al., 2019; Lee et al., 2019; Chen et al., 2020; Pan et al., 2020; Zhang et al., 2020; Ba et al., 2021; Chen et al., 2021; Fan et al., 2021; You et al., 2021).

**Table 5:** Primary Immunodeficiencies reported in HIV-negative children with T. marneffei infection.

Patients	Genetic defect	Mutation	Gender	Age	Residence	Detection methods	Extent of T.marneffei infection	Treatment and outcome
p1 (Lee et al., 2012) (Lee et al., 2019) (Lee et al., 2014)	STAT1, GOF	c.800C>T (p.A267V)	M	15y	Hong Kong, China	Fine-needle aspiration of the cervical lymph node for culture yielded T. marneffei	Disseminated	Liposomal amphotericin B for 6 weeks, followed by itraconazole prophylaxis with good clinical response
p2 (Lee et al., 2012) (Lee et al., 2019) (Lee et al., 2014)	STAT1, GOF	c.1074G>T (p.L358F)	F	7y	Hong Kong, China	BALF culture yielded T. marneffei	Disseminated	Lliposomal amphotericin B for 6 weeks, followed by itraconazole prophylaxis with good clinical response
p3 (Lee et al., 2012) (Lee et al., 2019) (Lee et al., 2014)	STAT1, GOF	c.863C>T (p.T288I)	F	7y	Hong Kong, China	Lymph node biopsy yielded T. marneffei	Disseminated	Treated with itraconazole with good response, died of massive pulmonary hemorrhage at 16 years old
p4 (Lee et al., 2019) (Yuen et al., 1986)	STAT1, GOF	c.1170G>A (p.M390I)	M	10y	Hong Kong, China	Tissue from the neck ulcer and axillary lymph node for culture yielded T. marneffei	Disseminated	Intravenous amphotericin B and oral flucytosine for 3 months with good response
p5 (Chen et al., 2020)	STAT1, GOF	c.193G>A (p.D65N)	M	5y11m	China	NA	Lymphadenitis	Amphotericin B and voriconazole, alive
p6 (Chen et al., 2020)	STAT1, GOF	c.1053G>T(p.L351F)	F	9y11m	China	NA	Pulmonary	Itraconazole and amphotericin B, alive
p7 (Fan et al., 2021)	STAT1	NA	M	2y	Hunan, China	Bone marrow culture yielded T. marneffei, Lymph node biopsy (Right inguinal hernia) fungal spore structure, PAS(+)	Disseminated	Intravenous voriconazole for 2 weeks, amphotericin B for 3 weeks, oral itraconazole for 1 year
p8 (Chen et al., 2021)	STAT1, GOF	nt.859T > A (Y287N)	M	20y	China	Blood, bone marrow and sputum cultures yielded T. marneffei	Disseminated	Amphotericin B for 6 months, recovery
p9 (Lee et al., 2012)	STAT3, Hyper-IgE syndrome	c.1121A>G (p.D374G)	F	12m	China	Blood and bone marrow cultures yielded T. marneffei	Disseminated	Treated with itraconazole with good response
p10 (Ma et al., 2009)	STAT3, Hyper-IgE syndrome	NA	M	10y	Hong Kong, China	Sputum and abscess fluid cultures yielded T. marneffei	Pulmonary	Treated with amphotericin B, died of respiratory failure due to rapid disease progression
p11 (Fan et al., 2018)	STAT3, Hyper-IgE syndrome	c.1593A>T (p.K531N)	M	13y	Guangzhou, China	BALF culture yielded T. marneffei	Disseminated	Treated with amphotericin B, voriconazole for 2 weeks and itraconazole orally for 2 months with good response
p12 (Pan et al., 2020)	STAT3, Hyper-IgE syndrome	c.1673G>A (p.G558D)	M	37m	Guangxi, China	The colon biopsy showed a large number of fungal spores, liver tissue revealed numerous intracellular yeast-like or sausage-like cells, bone marrow culture confirmed T.marneffei	Disseminated	Intravenous voriconazole and antibiotics for 10 days and oral voriconazole for 7 months, recovery
p13 (Zhang et al., 2020)	STAT3, Hyper-IgE syndrome	c.92G>A (p.R31Q)	M	34y	Zhejiang, China	mNGS of BALF confirmed T. marneffei (readers 566), cultures of BALF and the endobronchial biopsied tissue mass yielded T. marneffei	Pulmonary	Itraconazole, recovery
p14 (Fan et al., 2021)	STAT3, Hyper-IgE syndrome	NA	F	2y	Hunan, China	Cultures of bone marrow, sputum and BALF yielded T. marneffei	Disseminated	Amphotericin B for 2 days (discontinued due to liver dysfunction), Intravenous voriconazole for 4 days, give up and died
p15 (Lee et al., 2019) (Du et al., 2019)	CD40L(TNFSF5)	g.IVS1+1G>A	M	29m	China	Cervical lymph node and endobronchial biopsy yielded T. marneffei; cultures of blood, nasal secretions, throat swab and sputum yielded T. marneffei	Disseminated	Treated with voriconazole for 4 months and subsequent recurrence treated with voriconazole, with good response
p16 (Kamchaisatian et al., 2006)	CD40L deficiency	Complex mutation in exon 5	M	14m	Northeastern Thailand	Throat swab, sputum, blood and bone marrow cultures grew T. marneffei	Disseminated	Treated with amphotericin B for 21 days, followed by itraconzole for 10–12 weeks
p17 (Kamchaisatian et al., 2006)	CD40L deficiency	NA	M	1y	Northern Thailand	Lymph node tissue culture yielded T. marneffei	Pulmonary disease and lymphadenopathy	Treated with amphotericin B for 21 days, followed by itraconzole for 10–12 weeks
p18 (Sripa et al., 2010)	CD40L deficiency	NA	M	3y	Thailand	T. marneffei infection of the sputum	Pulmonary	Itraconazole, good response
p19 (Du et al., 2019)	CD40L deficiency	g.IVS1-3T>G	M	2y	Hunan,China	Blood culture and hepatic biopsy showed T. marneffei	Disseminated	Treated with amphotericin B, died of multi-organ failure
p20 (Li et al., 2018)	CD40L deficiency	NA	M	14m	Jiangxi,China	Blood culture yielded T. marneffei	Disseminated	Treated with itraconazole for 2 weeks and improved
p21 (Du et al., 2019)	CD40L deficiency	IVS3 + 1G>A	M	2y11m	China	Bone marrow culture yielded T. marneffei	Disseminated	Responded effectively to anti-fungal therapy
p22 (Du et al., 2019)	CD40L deficiency	IVS1-1 G > A	M	2y3m	China	Blood culture yielded T. marneffei	Disseminated	Lost to follow-up
p23 (Du et al., 2019)	CD40L deficiency	IVS4 + 1G>C	M	3y	China	Bone marrow culture yielded T. marneffei	Disseminated	Responded effectively to anti-fungal therapy
p24 (Du et al., 2019)	CD40L deficiency	Large fragment deletion including exon 4 and 5	M	13y7m	China	Blood culture yielded T. marneffei	Disseminated	Responded effectively to anti-fungal therapy
p25 (Lee et al., 2019)	IFNGR1	c.182dupT (p.V61fs69)	F	5m	Northern Thailand	NA	Disseminated	Die
p26 (Lee et al., 2019)	IFNGR1	c.182dupT (p.V61fs69)	M	12m	Northern Thailand	Blood culture yielded T. marneffei	Disseminated	Treated with amphotericin B for 6 weeks with good response, followed by itraconazole prophylaxis
p27 (You et al., 2021)	CARD9, compound heterozygote	c.440T>C (p.L147P), c.586A>G (p.K196E)	M	5y	Chongqing,China	Bone marrow smear identified T. marneffei infection, ascites culture yielded T. marneffei	Disseminated	Treated with amphotericin B and voriconazole, died of multiple organ failure
p28 (Ba et al., 2021)	CARD9, compound heterozygote	c.1118G>C (p.R373P), c.610C>T (p.R204C)	M	7m	Guangzhou, China	Blood culture and mNGS of blood confirmed T. marneffei (readers 248)	Disseminated	Voriconazole, good response

y, year; m, month; BALF, bronchoalveolar lavage fluid; mNGS, metagenomic next-generation sequencing; GOF, gain of function; M, male; F, female; NA, not available.

The median age of patients detected with *T. marneffei* reported previously was 3 years, ranging from 5 months to 34 years. Most of them were Chinese (82%, 23/28), and the others were from Thailand. Twenty patients (71%) had disseminated *T. marneffei* disease involving blood or bone marrow, lungs, colon, skin, lymph nodes, and liver. The immunodeficiency genes included *CD40LG* (35%), *STAT1* (29%), *STAT3* (22%), *CARD9* (7%) and *IFNGR1* (7%). Comparisons between our cohort and previously reported gene spectra are shown in **Figure 1B**.

A total of 46 positive clinical samples from these 28 patients were collected (**Figure 5**). *T. marneffei* was mainly detected by cultures (78.2%) of blood, bone marrow, sputum, BALF, tissue and so on. Histopathological staining of the biopsy tissue was followed (8.7%), including lymph node, liver, colonic mucosa and bone marrow samples. Only two patients were detected to have *T. marneffei* by mNGS (4.3%), 566 reads in BALF (Zhang et al., 2020) and 248 reads in blood (Ba et al., 2021). The detection method of *T. marneffei* in the remaining four samples (8.7%), including endobronchial biopsy, lymph node biopsy and hepatic biopsy, was not available.

Nearly half of the patients (12/28) were treated with amphotericin B, followed by itraconazole or voriconazole. Other patients were treated with itraconazole (5/28), voriconazole (2/28), or amphotericin B (3/28) alone. Only one patient received intravenous amphotericin B and oral flucytosine. The treatments of five patients were unknown. Except for one patient lost to follow-up, most patients (21/28) had improved symptoms with effective antifungal therapy. Six patients (6/28) died. Two patients died of multiorgan failure, one patient died of respiratory failure due to rapid disease progression, one patient died of massive pulmonary hemorrhage at 16 years old, and the other two patients died of unknown reason.

## 4 Discussion

*T. marneffei* is an opportunistic infectious pathogen. Inhalation of conidia is the primary route of infection, which are subsequently phagocytosed by alveolar macrophages, and then *T. marneffei* disseminates to the reticuloendothelial system, causing systemic infection when the host immune response is suppressed (Supparatpinyo et al., 1994). Therefore, patients susceptible to *T. marneffei* are usually immunosuppressed. Fungal infections in children with IEI are a growing concern.

T-cell-mediated immunity plays a central role in the immune defense mechanism against *T. marneffei* infection. Congenital athymic mice developed severe pulmonary and disseminated systemic Penicillium disease (Kudeken et al., 1996; Kudeken et al., 1997). A literature review showed that CD40 ligand deficiency was the most common type of immune deficiency in *T. marneffei* infection. CD40L mediates the interaction between T cells and different cells through ligation with its receptor CD40 (van Kooten and Banchereau, 2000; Erdos et al., 2005; Du et al., 2019). CD40 ligand-deficient patients suffered from opportunistic infections, especially *Pneumocystis jiroveci* and *Cryptosporidium parvum* infections. Recently, Pamela P. Lee discussed endemic mycoses in IEI, in which she highlighted *T. marneffei* infection in CD40 ligand deficiency in Southeast Asia (Lee and Lau, 2017).

Intrinsic and innate immunodeficiencies associated with *T. marneffei* infection have also been reported, such as *STAT1* and *CARD9*. Caspase recruitment domain–containing protein 9 (CARD9) is an adaptor molecule in the cytosol of myeloid cells required for the induction of T-helper cells producing interleukin-17 (Th17 cells), and it can effectively integrate the recognition signals of various natural immune receptors and plays an important role in antifungal immunity (Drummond et al., 2011; Salazar and Brown, 2018; You et al., 2021). Signal transducer and activator of transcription (STAT) proteins are critical transcription factors for the appropriate regulation of cellular responses to interferons, cytokines, growth factors, and hormones. There is tight regulation of their function *via* several mechanisms, and STAT1 and STAT3 play a central role (Villarino et al., 2015). A consistent immunophenotype in GOF *STAT1* and HIES patients is impaired development of Th17 lymphocytes, which could be the reason for their susceptibility to chronic mucocutaneous candidiasis or invasive fungal infection (Stark and Darnell, 2012; Olbrich and Freeman, 2018; Danion et al., 2020). This could explain the *T. marneffei* infection of P4 with *STAT3* and P5 with *STAT1* mutation.

It is noteworthy that the *IL12RB1* gene was first reported to be associated with *T. marneffei* infection in our cohort. *T. marneffei* infection has previously been reported in children with *IFNGR1* gene mutations. Both the *IL12RB1* and *IFNGR1* genes are involved in Mendelian susceptibility to mycobacterial disease (MSMD). Susceptibility to nontuberculous mycobacteria (NTM) infection, talaromycosis, histoplasmosis, cryptococcosis, melioidosis, and nontyphoidal salmonellosis has been reported in IFN-γ knockout mice or in patients with IFN-γ signaling defects, such as MSMD (Erb et al., 1999; Tang et al., 2010; Lee et al., 2013; Chi et al., 2016). A high prevalence of anti–IFN-γ autoantibodies is the major cause of severe *T. marneffei* infections in HIV-negative adults in China, which suggests that IFN-γ has a role in combating this fungal infection in humans (Guo et al., 2020). Human and mouse macrophages can control *T. marneffei* growth and kill intracellular yeast cells when activated by T-cell-derived cytokines (Cogliati et al., 1997; Sisto et al., 2003). IFN-γ is essential in the control of intracellular pathogens, such as Mycobacterium tuberculosis and fungi (Cooper et al., 1993). The fungicidal activity of *T. marneffei* yeast by macrophages could be enhanced by IFN-γ *via* stimulation of macrophages, which involves the nitric oxide (NO)-mediated killing system (Cogliati et al., 1997; Kudeken et al., 1998). In our study, three children were confirmed to have MSMD, including *IL12RB1* gene deficiency (P1, P2) and *IFNGR1* deficiency (P3). They had Mycobacterium tuberculosis and *T. marneffei* infections simultaneously. The clinical manifestations of P1 were the most serious, with recurrent fever, weight loss, severe anemia, splenomegaly and lymphadenopathy, requiring prolonged intensive antifungal treatment, as well as concomitant anti-Mycobacterium treatment.

Recently, mNGS has been successfully applied in the diagnosis of disseminated *T. marneffei* infection. The traditional standard for infection diagnosis relies on culture, histopathological staining and microscopy. Studies have shown that the combination of quantitative polymerase chain reaction (qPCR) and serum GM detection can be a valuable tool for the diagnosis of *T. marneffei* infection (Li et al., 2020; Wei et al., 2021). Patients with fungemia often have high GM results, such as P1, P2, P5 and P6. The measurement of BALF-GM is likely to be a useful tool for diagnosing invasive aspergillosis (Guo et al., 2010); however, its role in diagnosing *T. marneffei* has not been well established. mNGS is a new diagnostic technology to sequence all biological genomes in various clinical samples (Voelkerding et al., 2009). Compared with culture-based methods, mNGS showed obvious advantages with respect to high detection efficiency and quick speed. The detection accuracy of microorganisms, along with the positive rate, was higher in mNGS (Tsang et al., 2021; Zhou et al., 2021), which provides a faster and more accurate diagnostic method in clinical practice. The diagnostic efficiency analysis revealed that mNGS had a high diagnostic sensitivity of 100% and specificity of 98.7%. The significantly high positive likelihood rate indicated high accuracy of mNGS in determining *T. marneffei*, and the significantly low negative likelihood rate predicted that negative mNGS results could exclude *T. marneffei* infection. However, there was no significant difference between the two methods (p=0.25). Our study was a retrospective study, and there could be a risk of bias, which may originate in the relatively few patients enrolled, data collection and incomplete clinical data.

In clinical practice, the rapid diagnosis of *T. marneffei* infection in patients with IEI is of great importance. Disseminated *T. marneffei* infection may run a rapidly progressive course and be life-threatening without timely and effective antifungal therapy. However, cultures are quite time consuming (Cao et al., 2019), with a relatively limited positive rate due to the difficulty in cultivating slow-growing and fastidious microbes (Mo et al., 2002), which results in delayed diagnosis and increased mortality. A few studies have shown that mNGS has marked advantages over conventional methods for pathogenic diagnosis, particularly opportunistic pathogens and mixed infections in patients with IEI (Parize et al., 2017; Tang et al., 2021). For novel, rare, and treatment-refractory infectious diseases and for patients with immunocompromising disease, mNGS can significantly improve the pathogen detection rate and can be used as the front-line detection method (Cao et al., 2020). Unlike previous studies, mNGS was the main methodology in our cohort. mNGS can detect *T. marneffei* in a variety of samples, while traditional culture methods are often negative. In addition, the detection period of *T. marneffei* by mNGS was approximately 26 hours. Since infection can progress rapidly in children with immunodeficiency disease, pathogens should be identified as soon as possible using mNGS, and treatment can be initiated in time. Immunodeficient children may benefit from rapid detection of pathogens by mNGS.

## 5 Conclusion

*T. marneffei* is an opportunistic pathogen, suggesting potential immune impairment in infected individuals. We reported two cases of *IL12RB1* mutation in children infected with *T. marneffei*, extending the immunodeficiency spectrum of *T. marneffei* infection. For IEI patients with *T. marneffei* infection, we highlight the application of mNGS in the clinical diagnosis. mNGS is proposed as an important adjunctive diagnostic approach for rapidly identifying pathogens in complex and severe infections.

## Data availability statement

The datasets presented in this study can be found in online repositories. The names of the repository/repositories and accession number(s) can be found below: NCBI repository, the accession number is PRJNA860094.

## Ethics statement

The studies involving human participants were reviewed and approved by the ethics committee of Children’s Hospital of Fudan University. Written informed consent to participate in this study was provided by the participants’ legal guardian/next of kin. Written informed consent was obtained from the minor(s)’ legal guardian/next of kin for the publication of any potentially identifiable images or data included in this article.

## Author contributions

LL and BS contributed to conceptualizing and designing the study, conducting the investigation, and drafting the manuscript. JH contributed to conceptualizing and designing the study, conducting the investigation, and reviewing and revising the manuscript. XW contributed to conceptualizing the study and reviewing and revising the manuscript. JS contributed to supervising the methodology and reviewing the manuscript. WW, WY, MY, XH, and QZ contributed to collecting clinical data, conducting the investigation, carrying out the initial analyses, and reviewing the manuscript. YW and DL contributed to designing the methodology, conducting the laboratory experiments, and reviewing the manuscript. All authors approved the final manuscript as submitted and agreed to be accountable for all aspects of the work.

## Acknowledgments

We gratefully acknowledge Meili Shen from Dinfectome Inc., Nanjing, China, who helped with the bioinformatics analysis.

## Conflict of interest

The authors declare that the research was conducted in the absence of any commercial or financial relationships that could be construed as a potential conflict of interest.

## Publisher’s note

All claims expressed in this article are solely those of the authors and do not necessarily represent those of their affiliated organizations, or those of the publisher, the editors and the reviewers. Any product that may be evaluated in this article, or claim that may be made by its manufacturer, is not guaranteed or endorsed by the publisher.
